# Platelets as Mediators of Thromboinflammation in Chronic Myeloproliferative Neoplasms

**DOI:** 10.3389/fimmu.2019.01373

**Published:** 2019-06-14

**Authors:** Cecilia P. Marin Oyarzún, Paula G. Heller

**Affiliations:** Department of Hematology Research, National Scientific and Technical Research Council (CONICET), Institute of Medical Research (IDIM) “Dr. Alfredo Lanari”, University of Buenos Aires, Buenos Aires, Argentina

**Keywords:** chronic myeloproliferative neoplasms, platelets, JAK2, thrombosis, inflammation, innate immunity, bleeding

## Abstract

Chronic myeloproliferative neoplasms (MPN) are stem cell disorders driven by mutations in *JAK2, CALR*, or *MPL* genes and characterized by myeloid proliferation and increased blood cell counts. They encompass three closely related conditions, including essential thrombocythemia, polycythemia vera, and primary myelofibrosis. Elevated levels of cytokines released by clonal and non-clonal cells generate a chronic proinflammatory state that contributes to disease pathogenesis. Thrombosis represents the most common cause of morbidity and mortality in MPN, although paradoxically, patients may also present with a bleeding diathesis. The mechanisms leading to thrombosis are complex and multiple and include increased blood cells together with qualitative abnormalities of red cells, leukocytes, and platelets that favor a prothrombotic activated phenotype. The functional interplay between blood cells, the clotting cascade, and dysfunctional endothelium contributes to hypercoagulability and this process is perpetuated by the effect of inflammatory cytokines. In addition to their well-known function in hemostasis, platelets contribute to innate immunity and inflammation and play a key role in MPN thromboinflammatory state. *In vivo* platelet activation leads to platelet aggregate formation and exposure of adhesion molecules which favor their interaction with activated neutrophils and monocytes leading to circulating platelet-leukocyte heterotypic aggregates. Platelets are recruited to the activated endothelium further enhancing the reciprocal activation of both cell types. Crosstalk between activated cells drives cytokine production, further fuelling the self-reinforcing thromboinflammatory loop. In addition, MPN platelets provide a procoagulant scaffold which triggers the coagulation cascade and platelet-derived microparticles amplify this response. Markers of platelet, leukocyte, endothelial and coagulation activation are increased in MPN patients although prospective studies are required to determine the potential value of these parameters for identifying patients at increased thrombotic risk. Thrombosis remains the main complication of MPN patients, with a high risk of recurrence despite adequate cytoreductive and antithrombotic treatment. Deeper insight into the mechanism favoring thrombosis development in this setting may lead to novel therapeutic approaches for MPN thrombosis. Considering the critical role of inflammation in the vascular risk, concomitant targeting of inflammatory pathways could potentially impact on primary or secondary prevention strategies.

## Introduction

Philadelphia-negative chronic myeloproliferative neoplasms (MPN) are clonal hematopoietic stem cell disorders characterized by excessive production of myeloid progenitors and mature blood cells. They comprise three closely related disorders, including essential thrombocythemia (ET), which is characterized by megakaryocyte proliferation and thrombocytosis, polycythemia vera (PV), which is defined by predominant erythroid expansion and increased red blood cells, frequently associated with high leukocyte and platelet counts, and primary myelofibrosis (PMF), featured by increased numbers of dysplastic megakaryocytes and granulocyte progenitors together with variable degrees of bone marrow fibrosis (1). Hyperactivation of JAK2-signaling is a common feature in MPN pathogenesis and is driven by mutations in three genes, including *JAK2, CALR*, and *MPL*. The *JAK2*V617F mutation is the most frequent molecular abnormality and may be found in over 95% of patients with PV and 50–60% of those with ET and PMF (1, 2). Defects in calreticulin (*CALR*) represent the second most frequent abnormality, which can be detected in 20–30% of ET and PMF patients (1, 2). Calreticulin mutants interact abnormally with the Mpl receptor leading to its activation and persistent JAK2 signaling (3). Finally, *MPL* mutations can be found in a low proportion (1–10%) of ET and PMF patients and generate constitutive receptor activation. None of the above-mentioned mutations are detected in 10–25% of ET and PMF cases, so-called triple-negative patients (1, 2).

Thrombosis is the main cause of morbidity and mortality in MPN and develops in around 20–35% of patients with PV, 15–30% in ET, and 10–15% in PMF (4, 5). Arterial thrombosis accounts for 60–70% of all vascular complications, and include stroke, cardiovascular events, and peripheral artery disease, whereas venous events include deep venous thrombosis and pulmonary embolism, but may also occur at unusual sites, such as the splachnic circulation. Indeed, MPN are the most frequent underlying disorders leading to Budd-Chiari syndrome and non-cirrhotic portal vein thrombosis, which may develop even in the absence of overt MPN (4, 5). A population-based study on the causes of death in MPN patients showed that cardiovascular and cerebrovascular disease accounted for high risk of death at all ages, particularly in younger patients. The most common cause of death was cardiovascular disease in patients with PV and ET, whereas patients with PMF had an increased probability of dying from hematologic malignancies (6). In addition to large-vessel thrombosis, transient platelet aggregates may clog small vessels and lead to microvascular disturbances, such as erythromelalgia and visual abnormalities, seen typically, but not exclusively, in ET (7). Paradoxically, MPN patients may also suffer from bleeding complications, which also substantially contribute to morbidity in these disorders.

## Thrombotic Risk Factors

The main risk factors for thrombosis include age over 60 years and a previous history of thrombosis. According to the presence or absence of these factors, patients are stratified into low- or high-risk groups in order to guide treatment recommendations and the use of cytoreductive therapy (1). More recently, the IPSET-thrombosis model, which includes cardiovascular risk factors and the *JAK2*V617F mutation, has been proposed to better predict the thrombotic outcome in ET (8), although this score has not been yet incorporated into clinical practice. The influence of the *JAK2*V617F mutation in the thrombotic risk has been established by several studies (1, 9) and confirmed by a meta-analysis, which revealed a two-fold increase in vascular events (10). Interestingly, individuals harboring *JAK2*V617F-positive clonal hematopoiesis of indeterminate potential, in the absence of overt MPN, have an increased thrombotic risk, highlighting the relevance of the *JAK2* mutation in the thrombotic predisposition (11, 12). Conversely, *CALR*-positive patients are at lower risk of thrombosis (1).

## Pathogenesis of MPN Thrombosis

The pathogenesis of thrombosis in MPN is multifactorial and results from the complex interplay among blood cells, the endothelium and the clotting system. Increased numbers of red cells, leukocytes and platelets coupled to qualitative abnormalities that favor a prothrombotic phenotype contribute to the hypercoagulable state (4, 5). Hyperviscosity due to increased red cell mass clearly plays a role in the thrombotic predisposition of PV and, moreover, PV red cells display enhanced adhesion to endothelial laminin (13). In addition, high hematocrit favors platelet margination and accumulation at sites of vascular injury (14). In recent years, growing evidence has highlighted the key role of leukocytes in the prothrombotic state and leukocytosis has been shown to be an independent risk factor for thrombosis (15). In addition to increased numbers, there is evidence of *in vivo* neutrophil activation, as revealed by CD11b expression and elevated elastase and myeloperoxidase in circulation (16, 17), and of monocyte activation, as shown by elevated CD25 (18). Another element contributing to the thrombotic tendency involves endothelial dysfunction, which renders a pro-adhesive and proinflammatory surface, favoring leukocyte and platelet tethering and activation (4, 5).

It is currently well-established that the MPN clone induces a systemic inflammatory response, reflected by elevated levels of a wide spectrum of proinflammatory cytokines, such as IL-6, IL-1, IL-8, and TNFα (19). Inflammation and hemostasis are closely connected processes and, recently, the link between the innate immune system and coagulation as a host defense strategy against pathogens has led to the concept of immunothrombosis (20). Dysregulation of this mechanism may drive vascular disease and contribute to arterial and venous thrombosis in several disease conditions (20). Emerging work highlights the contribution of chronic inflammation to MPN hypercoagulable state, as demonstrated by the association of elevated C-reactive protein and thrombosis (5, 21). Inflammatory mediators favor the activation of both malignant and non-malignant blood cells, induce microparticle generation and elicit vascular damage, fuelling the thrombotic process (5).

## Role of Platelets in MPN Thrombosis

### Platelet Activation

Platelets are essential players in MPN thrombosis. Their role in this process and interplay with other elements of the procoagulant network represents the focus of this review. Both increased platelet numbers and *in vivo* activation may be involved in the prothrombotic phenotype. However, considerable controversy exists regarding the role of thrombocytosis in the thrombotic risk, as no correlation has been shown between platelet counts and vascular complications (22). Furthermore, extreme thrombocytosis (>1,000–1,500 × 10^9^/L) is associated with increased bleeding rather than thrombosis. Indeed, ET patients with extreme thrombocytosis, in the absence of leucocytosis, carry a lower thrombotic risk (23). Nonetheless, reduction in platelet counts by cytoreductive therapy is useful at preventing thrombotic and bleeding complications (24–26) and current recommendations suggest that the goal of cytoreduction is to target the platelet count at <400 × 10^9^/L (27). Notwithstanding controversy regarding the role of high platelet counts, there is unequivocal evidence supporting the contribution of platelet activation to the procoagulant state. *In vivo* platelet activation has been demonstrated in ET, PV and also in PMF (17, 28–31), as revealed by the finding of platelet activation markers, such as surface and soluble P-selectin and CD40L (17, 28, 29), raised β-thromboglobulin, platelet factor 4 and PDGF in plasma (32, 33) and urinary TXB2 metabolite as a reflection of thromboxane biosynthesis (30).

**Figure 1:**
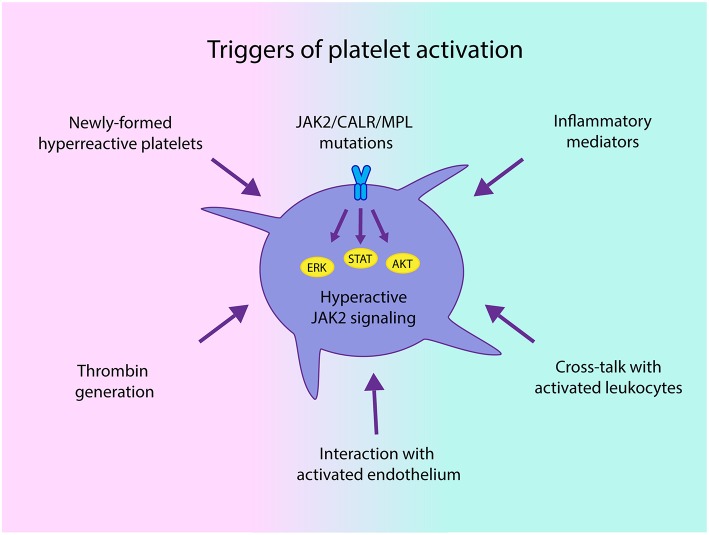
Triggers of platelet activation in chronic myeloproliferative neoplasms (MPN). Both intrinsic abnormalities derived from the MPN clone, such as JAK2-dependent hyperactivation of signaling pathways and hyperreactive newly-formed platelets, as well as extrinsic signals driven by enhanced interaction with activated leukocytes and endothelial cells and soluble mediators, including classical platelet agonists, such as thrombin generated by the hypercoagulable state and inflammatory factors may all converge to trigger platelet activation in MPN.

The mechanisms leading to platelet activation involve both intrinsic platelet abnormalities derived from disturbed hematopoietic stem cell function and linked to driver mutations that lead to hyperactive JAK2-dependent signaling (34), and extrinsic factors, such as cellular interaction with activated leukocytes, endothelial cells and soluble mediators, which may trigger activation of platelets derived not only from clonal cells, but also from megakaryocytes not involved in the malignant clone (Figure 1). In addition, increased platelet turnover leading to higher proportion of newly-formed platelets, which are known to display enhanced platelet reactivity, may also contribute to the hyperactivated state (29, 35). Previous data suggest that changes in megakaryocyte gene expression profile might give rise to circulating platelets with an altered hemostatic or inflammatory function in infectious or inflammatory conditions (36). Whether changes in megakaryocyte transcriptome may be associated with a similar phenotype in MPN platelets remains an intriguing possibility. In this regard, downregulation of several genes involved in thrombin signaling and platelet activation has been demonstrated in *CALR*- vs. *JAK2*V617F-positive patient samples, correlating with lower thrombotic predisposition in the former (37).

Among MPN disorders, platelet hemostatic abnormalities have been most thoroughly studied in ET. Despite the presence of basal platelet activation, conflicting results have been published regarding the *ex vivo* response to classical agonists, such as ADP and TRAP. Whereas, some studies showed normal or enhanced response to stimuli (17), others described an impaired hemostatic function, as shown by reduced P-selectin, CD63 expression and PAC-1 or fibrinogen binding triggered by classical agonists (38, 39). Similarly, light transmission aggregometry may reveal spontaneous platelet aggregation together with impaired response to different agonists, particularly ADP and epinephrine (40), although it has been suggested that the *in vitro* aggregation defect may be partly due to a laboratory artifact (41) or depend on analytical conditions (42). *In vivo* release of platelet granule contents due to spontaneous activation and secondary storage pool deficiency may explain the platelet function defect found *ex vivo*. Coexistence of platelet activation and dysfunction may contribute to the paradoxical occurrence of both thrombotic and bleeding complications. In addition, adsorption of high molecular weight multimers of von Willebrand factor (vWF) to platelet GPIbα leading to loss of large vWF multimers and acquired von Willebrand disease contributes to the bleeding diathesis (43).

**Figure 2:**
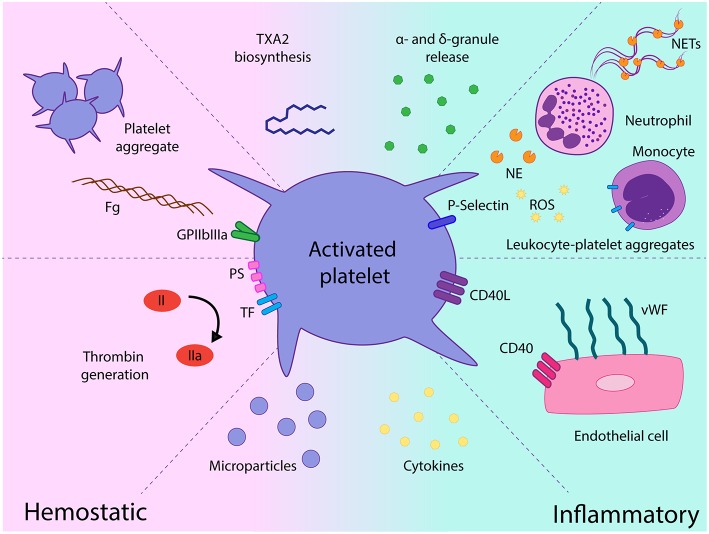
Role of platelets as mediators of hemostatic and proinflammatory responses in chronic myeloproliferative neoplasms (MPN). Platelet activation leads to increased GPIIbIIIa activation and platelet aggregate formation, platelet procoagulant response promotes thrombin generation on the platelet surface and platelet-derived microparticles futher fuel coagulation. Release of a miriad of α- and dense granule hemostatic and inflammatory mediators may contribute to the prothrombotic and proinflammatory loop. Enhanced interaction with leukocytes mediated by P-selectin leads to leukocyte-platelet heterotypic aggregates and may trigger several leukocyte responses, such as release of proteases, production of reactive oxygen species, and expression of tissue factor. Exposure of CD40L favors platelet recruitment and adhesion to the endothelium which, in turn, elicits endothelial cell activation, featured by vWF release from Weibel-Palade bodies. Elevated cytokines in the MPN milieu may promote platelet activation and, reciprocally, platelets may represent a potential source of inflammatory cytokines and chemokines. TXA2, thromboxane A2; Fg, fibrinogen; TF, tissue factor, PS phosphatidylserine; NE, neutrophil elastase; ROS, reactive oxygen species; NETs, neutrophil extracellular traps; vWF, von Willebrand factor, EC, endothelial cell.

Activated platelets mediate several functional responses which contribute to MPN prothrombotic state. In addition to the classical platelet hemostatic properties, growing body of evidence over the last decade highlights the relevance of alternative platelet functions, such as their role as effectors of inflammation and essential players in the innate immune response (44). The contribution of platelets as mediators of thromboinflammatory responses in MPN and their interplay with other components of the prothrombotic and proinflammatory circuit is discussed below and summarized in Figure 2.

### Platelet-Leukocyte Cross-Talk

Platelet interaction with leukocytes is central to MPN prothrombotic scenario. Platelets bind to both neutrophils and monocytes through adhesion molecules such as P-selectin, which recognizes the PSGL-1 counterreceptor, and through GPIbα and GPIIbIIIa (via fibrinogen), both of which engage CD11b/CD18 (Mac-1). In accordance to elevated P-selectin, increased levels of platelet-neutrophil and platelet-monocyte aggregates have been shown in circulation in ET (17, 28), PV (28), and PMF (31). Crosstalk between platelets and leukocytes triggers the reciprocal activation of both cell types, contributing to the activated phenotype.

Several leukocyte functional responses are enhanced in MPN neutrophils, including the release of intracellular proteases, such as elastase and catepsin G, which activate platelets and trigger the clotting cascade by inactivating coagulation inhibitors (16). In addition, MPN neutrophils produce high levels of reactive oxygen species (45, 46), which lead to endothelial injury and modify coagulation factors. Besides these classical neutrophil functions, more recently, neutrophils have been shown to release extracellular traps (NETs), which are networks of DNA, histones and granular components which promote thrombus formation (47). Although deregulated NET formation underlies several prothrombotic conditions (47), the role of NETs in MPN remains controversial. Whereas, MPN neutrophils seldom undergo spontaneous NETosis *ex vivo* (11, 46), their response to stimuli was shown to vary according to the experimental setting. In this regard, enhanced response to ionomycin was shown in one study (11), while NETosis triggered by inflammatory cytokines and PMA was normal or impaired in another study (46), suggesting differential response to diverse NET inducers. Considering that activated platelets are a known trigger for NET formation, it might be relevant to study platelet-induced NETosis in these conditions. Likewise, platelet cross-talk with monocytes may prime monocyte functions, including tissue factor expression (48), which is increased at baseline in MPN (17), and cytokine synthesis (49), which has been found to be constitutively upregulated in PMF monocytes (50), thus perpetuating MPN prothrombotic and proinflammatory loop.

### Platelet-Endothelial Interaction

The functional interplay between activated platelets and endothelial dysfunction plays an important role in the prothrombotic state. Evidence for endothelial activation in MPN is well-established, as reflected by elevated vWF antigen, soluble thrombomodulin and E-selectin (16, 29). Activated endothelial cells exhibit a pro-thrombotic phenotype which fosters platelet and leukocyte recruitment. Release of vWF from Weibel-Palade bodies tethers and activates platelets, leading to the surface translocation of platelet CD40 ligand (CD40L), which binds endothelial CD40. Cleavage of membrane CD40L generates a soluble fragment (sCD40L), which is increased in MPN plasma (29). Several factors contribute to endothelial activation in MPN, including interaction with blood cells, reactive oxygen species and inflammatory cytokines. Intriguingly, the *JAK2*V617F mutation has been detected in mature endothelial cells from selected organs, such as the spleen of PMF patients and the liver of PV patients with Budd-Chiari syndrome, suggesting the potential involvement of endothelial cells in the malignant clone (51, 52). Endothelial-like cells differentiated from MPN patient-derived induced pluripotent stem cells (53) and *JAK2*V617F-transduced HUVECs exhibit pro-adherent properties *in vitro* (54). Moreover, increased thrombus formation has been demonstrated in mouse models expressing *JAK2*V617F only in the endothelial compartment (54), overall suggesting that *JAK2*-mutant endothelial cells could contribute to the prothrombotic phenotype.

### Procoagulant Potential of Platelets

MPN patients show several laboratory abnormalities indicative of chronic low-grade activation of the clotting system, such as elevated thrombin–antithrombin complexes, prothrombin fragment 1 + 2 and D-dimer levels (16, 29). Platelets are endowed with coagulation factors and activated platelets expose phosphatidylserine on their membrane, providing a catalytic substrate for the assembly of coagulation complexes and thrombin generation. Indeed, increased baseline phosphatidylserine expression on the platelet membrane has been shown in some MPN patients and overall platelet procoagulant potential was increased, as revealed by the finding of elevated platelet-induced thrombin generation (55). In addition, MPN platelets express higher surface levels of tissue factor, which represents the main initiator of blood coagulation, further enhancing the procoagulant activity (56).

### Platelet-Derived Microparticles

Patients with ET harbor higher numbers of circulating microparticles of platelet, endothelial and leukocyte origin, with the former comprising the vast majority of the microparticle population (57). Remarkably, a subset of microparticles co-expressing platelet and endothelial markers were also detected in ET, suggesting their bilineage origin. Platelet-derived microparticles are rich in tissue factor and phospholipid-dependent procoagulant activity and may deliver platelet-derived cytokines and chemokines, thus amplifying platelet proinflammatory and procoagulant signals.

### Platelets as Immune Cells

Platelets play a key role in innate immunity and inflammation through their interaction with other immune cells and the release of proinflammatory mediators (44) and thereby participate in several disease conditions characterized by acute or chronic inflammation, such as infection, autoimmune disorders, and atherosclerosis. MPN patients display raised levels of a broad array of cytokines and chemokines in circulation, which are aberrantly secreted by multiple cell populations, including monocytes, neutrophils and hematopoietic stem cells (58). Activated platelets release pro-inflammatory chemokines stored in their α-granules, such as RANTES (CCL5) and platelet factor 4 (PF4) (CXCL4) and may undergo *de novo* cytokine synthesis following agonist-triggered RNA splicing, as shown for IL-1β. The potential contribution of platelets as a source of cyto/chemokines and inflammatory mediators in MPN has not been explored. Alternatively, platelet interaction with monocytes could deliver signals that upregulate monocyte proinflammatory gene expression, thus contributing to elevated cytokine secretion (50). Reciprocally, elevated proinflammatory cytokines might contribute to platelet activation in MPN. In this regard, IL-1β has been shown to foster hemostatic responses in normal platelets (59).

Patients with MPN carry a significant risk of second malignancies, including both solid tumors and lymphomas (60). Platelets promote tumor growth and invasiveness through several mechanisms, including the release of growth factors, cytokines, and regulators of angiogenesis. Moreover, thrombocytosis in solid tumors is associated with inferior survival supporting the role of platelets in tumor progression. On this basis, it is tempting to consider the possibility that elevated platelet counts could contribute to tumorigenesis in the setting of MPN second cancers (61). Furthermore, although the contribution of platelets to innate immunity has been more extensively studied, platelets also influence adaptative immune responses through their interaction with T-cells, NK-cells and dendritic cells (44). Platelet-coated tumors may evade NK destruction by inhibiting NK cytotoxicity (62). In this setting, elevated platelet counts might also have implications in tumor immune surveillance and immunoregulation.

### Relationship Between Platelet Activation and Clinical Features

Several factors account for the higher frequency of thrombosis observed in *JAK2*-positive ET patients, including higher hemoglobin and leukocyte counts, lower platelet counts and older age compared to *CALR*-positive and triple-negative patients. In addition, *JAK2*-positive patients display higher levels of platelet activation markers (17, 29, 56, 63), as well as leukocyte and endothelial activation and circulating microparticles (29, 56, 64), which may represent additional elements favoring thrombosis development in this subset. In addition, although sP-selectin and sCD40L were shown to correlate with a previous history of thrombosis in one study (29), currently, the value of platelet activation markers to estimate the vascular risk remains uncertain. Prospective studies assessing the role of platelet, together with leukocyte, endothelial and coagulation activation parameters in the same MPN cohort would be required to adequately address this issue and to establish whether one or more of these parameters might be useful to predict the risk of thrombosis in this setting.

On the other hand, despite the usefulness of cytoreductive therapy in preventing thrombosis development (24–26), controversy exists regarding its influence on platelet reactivity. Whereas, no difference in platelet activation was shown between patients with and without cytoreductive treatment in two different studies (16, 17), analysis of sequential samples of patients treated with hydroxyurea demonstrated a decrease in platelet-neutrophil aggregates (65) and this drug was shown to block P-selectin-triggered platelet-aggregate formation *in vitro* (65). Similarly, despite the relevance of JAK2-dependent signaling in MPN cellular abnormalities and the fact that the JAK1/2 inhibitor ruxolitinib reduces the thrombotic risk (66), no decrease in platelet activation markers was shown in patients treated with this drug (67). In another study, normalization of circulating platelet-derived microvesicles was noted in ruxolitinib spleen-responders (68), pointing out that the impact of JAK2 inhibitors in hemostatic parameters deserves further evaluation.

## Antiplatelet Therapy

The efficacy of low-dose aspirin for the prevention of thrombotic complications in MPN highlights the key role of platelets in this setting (69). Patients with extreme thrombocytosis (>1,000 × 10^9^/L) are at higher risk of bleeding under aspirin, mainly attributed to acquired von Willebrand disease. Screening for ristocetin cofactor activity and withholding aspirin therapy if <30% is suggested in this scenario (70). Current guidelines recommend aspirin for primary prevention in PV, high-risk ET and low-risk *JAK2*-mutated ET patients who have no contraindications for antiplatelet therapy (70). On the other hand, considering that aspirin treatment of *CALR*-positive low-risk ET patients does not reduce thrombosis and may actually increase bleeding, it is not routinely recommended for primary prophylaxis in this subgroup (71). Aspirin has also been shown to reduce thrombosis recurrence after both a first arterial or venous event (72). However, this risk remains high even after combined cytoreductive plus anticoagulant or antiplatelet therapy (73), highlighting the need for novel therapeutic antithrombotic strategies. Persistent thromboxane biosynthesis due to accelerated platelet production may lead to aspirin resistance in MPN (74). On this basis, a twice-daily schedule has been proposed for optimal platelet inhibition in patients with very high-risk features (70).

Statins have been shown to decrease the risk of both atherothrombotic events and venous thromboembolism. Among their pleiotropic actions, statins exert potent anti-inflammatory effects on the vascular wall and suppress platelet, endothelial and leukocyte activation (75). Due to these properties, statins might be relevant agents in MPN, although their role to prevent thrombotic events in this setting remains to be established (76).

## Concluding Remarks

Thrombosis remains the main complication of MPN patients, with a high risk of recurrence despite adequate cytoreductive and antithrombotic treatment (73). Multiple and concomitant factors converge to increase the thrombotic risk in MPN, although the relative role of each factor may vary in the individual patient. Although progress has been made in unraveling the mechanisms underlying the thrombotic predisposition, the role of platelet and cellular activation markers in identifying patients at increased risk of thrombosis has not been established and prospective studies are needed to address this issue. Finally, the impact of new therapies on platelet and cellular activation and the potential development of novel therapeutic approaches to manage MPN coagulopathy could contribute to thrombosis prevention and result in improved patient outcome. Considering the critical role of inflammation in the vascular risk, simultaneous targeting of inflammatory pathways could potentially impact on primary or secondary prevention strategies.

## Author Contributions

CM contributed to the writing of the article and prepared the figures. PH wrote the article. Both authors contributed to manuscript editing and final approval.

### Conflict of Interest Statement

The authors declare that the research was conducted in the absence of any commercial or financial relationships that could be construed as a potential conflict of interest.
